# *p53* and *cyclin G* cooperate in mediating genome stability in somatic cells of *Drosophila*

**DOI:** 10.1038/s41598-017-17973-z

**Published:** 2017-12-20

**Authors:** Fabienne E. Bayer, Mirjam Zimmermann, Patrick Fischer, Christian Gromoll, Anette Preiss, Anja C. Nagel

**Affiliations:** 1 Institut für Genetik, Universität Hohenheim, Stuttgart, Germany; 2 Present Address: Institute of Molecular Biology/CMBI, University of Innsbruck, Innsbruck, Austria; 3 Department of Medical Physics, Marienhospital Stuttgart, Stuttgart, Germany

## Abstract

One of the key players in genome surveillance is the tumour suppressor p53 mediating the adaptive response to a multitude of stress signals. Here we identify Cyclin G (CycG) as co-factor of p53-mediated genome stability. CycG has been shown before to be involved in double-strand break repair during meiosis. Moreover, it is also important for mediating DNA damage response in somatic tissue. Here we find it in protein complexes together with p53, and show that the two proteins interact physically *in vitro* and *in vivo* in response to ionizing irradiation. In contrast to mammals, *Drosophila* Cyclin G is no transcriptional target of p53. Genetic interaction data reveal that p53 activity during DNA damage response requires the presence of CycG. Morphological defects caused by overexpression of *p53* are ameliorated in *cycG* null mutants. Moreover, using a p53 biosensor we show that p53 activity is impeded in *cycG* mutants. As both p53 and CycG are likewise required for DNA damage repair and longevity we propose that CycG plays a positive role in mediating p53 function in genome surveillance of *Drosophila*.

## Introduction

Environmental or intrinsic stress like ionizing radiation (IR), reactive oxygen species or chemical agents, challenges genome integrity an organism has to cope with. Accordingly a variety of strategies have evolved to combat DNA damage that are remarkably conserved during evolution from unicellular organisms like yeast to multicellular organisms like flies or mammals (overview^1–3^). DNA double-strand breaks (DSBs) are a particular threat to chromosome stability and distribution. They are generally repaired by either non-homologous end joining which is error-prone, or by homologous recombination repair which may use a sister chromatid for high fidelity repair in the G2 phase of the cell cycle (overview^3,4^). Upon sensing of single or double-strand breaks by specific protein complexes, checkpoint kinases (ATM/ATR, Chk1/2) are recruited to the site of DNA damage. By phosphorylation of specific target proteins, for example variant histone H2A (γ-H2Av), they trigger the transmission and amplification of DSB signals to downstream repair proteins (overview^1,3,5^). Failure of effective DNA damage repair may lead to regulated cell death, i.e. apoptosis, or to cell transformation and eventually carcinogenesis.

One of the key players in the surveillance of genome stability is the tumour suppressor gene *p53*. The *p53* gene family is evolutionary conserved, mediating the adaptive response to a variety of stress signals that threaten cellular homeostasis (overview^6,7^). In general, p53 proteins serve three major outcomes to genotoxic stress: firstly cell cycle arrest to allow for timely DNA damage response, secondly activation of repair genes, and finally initiation of apoptosis in case of irrevocable damage^6,8,9^. Genotoxic stress results in the stabilization and activation of p53 protein, which itself acts as transcriptional activator of a number of target genes. Two prime examples of mammalian p53 target genes are mdm2 and cyclin G1 (Ccng1) that both function in the negative regulation of p53 activity. In concert they guide dephosphorylation of activated p53 (overview^10,11^). Moreover, the E3 ubiquitin-ligase Mdm2 provokes p53 proteasomal degradation (overview^12^).

In *Drosophila* a single *p53* gene exists, which controls genome stability by activating repair genes and apoptosis induction similar to its vertebrate counterparts^3,7,13^. For example, well established p53 targets in *Drosophila* are pro-apoptotic genes like *head involution defective (hid)* or *reaper (rpr)* ^13^. There are a number of striking differences however. Only recently, a functional analogue of *mdm2* in *Drosophila* named *Corp* has been identified, which acts as a negative regulator of p53 protein. Indeed, *Corp* is a transcriptional target of p53, and the two proteins have been shown to interact directly^14^. Moreover, in response to IR-stress the demethylase UTX acts as a specific epigenetic co-factor of p53 in the transcriptional upregulation of the DNA repair gene *Ku80*, but is not required for the p53-mediated expression of either *hid* or *rpr* ^15^. This implies that *Drosophila* p53 might recruit different co-factors to fulfil its specific activities in response to various stressors. Here we describe the role of Cyclin G (CycG) as a co-factor of *p53*-mediated genome stability in *Drosophila*. We have shown earlier that CycG is involved in DSB sensing and repair during meiosis^16^, and now find that it is also important for combating genotoxic stress in somatic tissue. Although *Drosophila* CycG is not a transcriptional target of p53 like its mammalian counterpart cyclin G1, it physically interacts with p53 and is essential for p53 mediated DNA damage response. We provide evidence that p53 activity is hampered in the absence of *cycG* suggesting that CycG and p53 function together in the process of DNA damage repair.

## Results

### Loss of CycG compromises transposon-induced DSB repair

The fact that *cycG* mutants are impeded in meiotic DSB repair^16^ prompted us to investigate its involvement in somatic DSB repair. To gain first insights we decided to employ the *Drosophila* P{*w* ^*a*^}-element based system, which allows to uncover DSB repair-defects after P-element transposon excision^17^. This elegant assay is based on a X-linked P-element P{*w* ^*a*^} that carries the *apricot* allele (*w* ^*a*^) of the *white (w)* gene. The *w* ^*a*^ allele is characterized by a *copia* insertion in an intron of *w*, decreasing the expression of the *w* gene. Thus flies with only one copy of *w* ^*a*^ are identified by yellow eye colour, whereas those with two copies have apricot-coloured eyes^17^. DSBs are induced by mobilizing the P{*w* ^*a*^}-element with help of the Δ2-3 transposase in male flies and female progeny is judged by eye colour (Supplementary Fig. S1). Depending on the repair mechanism, eye colour may be apricot (exact repair by homologous recombination), yellow (absent or defective repair like non-homologous end joining), or red (reflecting loss of the copia-element)^17^.

**Figure 1:**
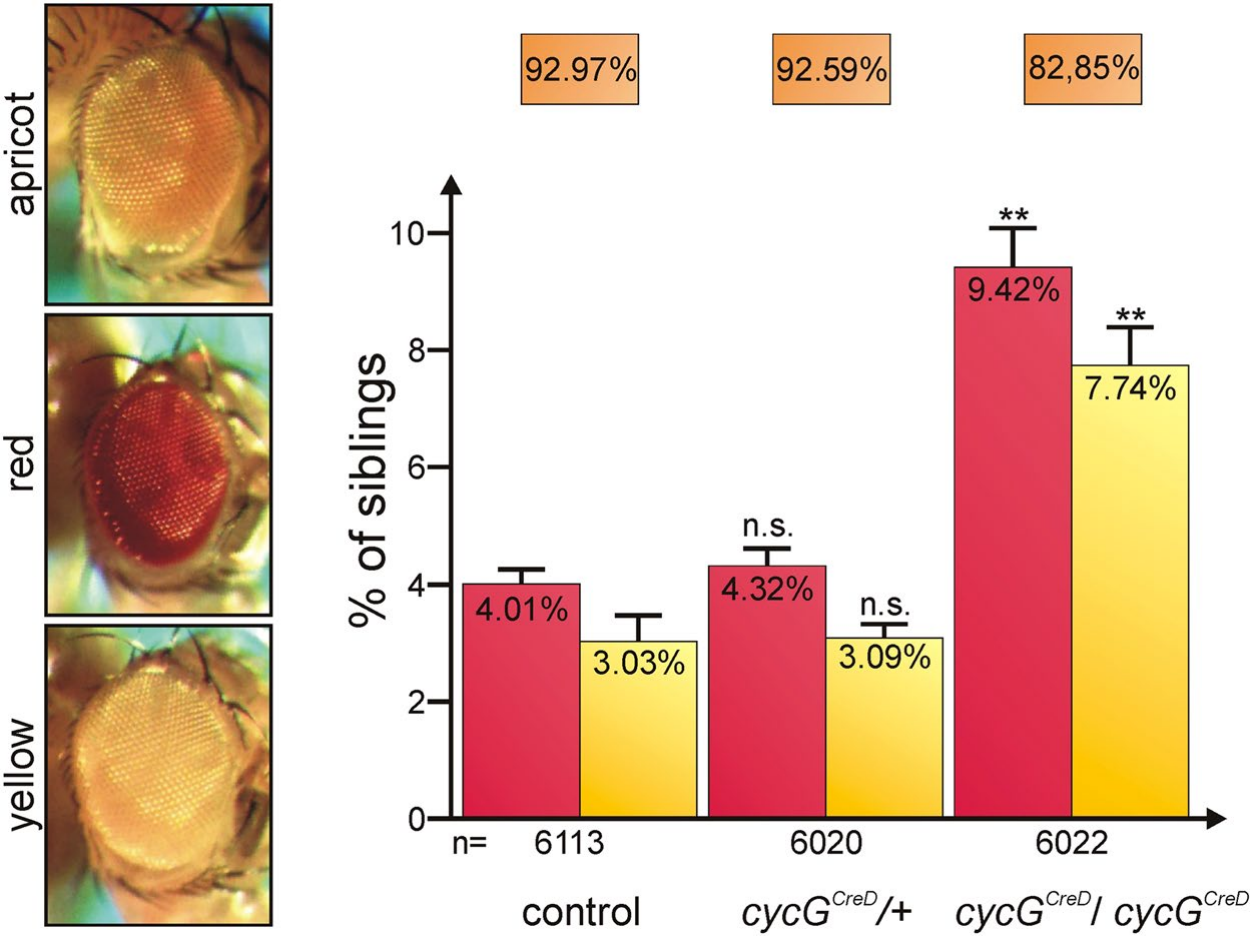
P-element based DSB repair assay with *cycG* ^*CreD*^ mutants. The P{*w* ^*a*^}-element was introduced in a Oregon-R control, a *cycG* ^*CreD*^ heterozygous or homozygous background, mobilized by transposase and backcrossed with P{*w* ^*a*^} (for details see Supplementary Fig. S1, and^17^). Eye colours were scored in the F_2_-female offspring; the fraction with apricot eye colour, red and yellow eye colour, respectively, was determined. The total number of analysed females (n) was 6113 for the control, 6020 for *cycG* ^*CreD*^/+ heterozygotes and 6022 for *cycG* ^*CreD*^ homozygotes. Error bars show standard error. Frequency of apricot, red and yellow eye coloured offspring was not significantly different between the control and the *cycG* ^*CreD*^ heterozygotes (n.s., p-values 0.34, 0.22 and 0.64 determined by Students T-test, respectively), whereas the respective fractions of the *cycG* ^*CreD*^ homozygotes varied significantly from control (**p-values 0.0005, 0.0022 and 0.010, respectively).

To employ this assay we made use of the *cycG* ^*CreD*^ null allele being deficient for *white* ^18^, combined it with the P{*w* ^*a*^}-element and transposase and analysed the female offspring. Heterozygous *cycG* mutants were similar to control flies: about 93% of the progeny had apricot-coloured eyes, whereas about 3% failed to repair DSBs properly (yellow-coloured eyes) (Fig. 1 and Supplementary Fig. S1). In contrast, only 83% of the homozygous *cycG* mutant female progeny had apricot-coloured eyes, whereas the percentage with either red- or yellow-coloured eyes was significantly increased with more than twofold of the controls or the heterozygotes (Fig. 1 and Supplementary Fig. S1). This indicates that in the absence of CycG, somatic repair of double-strand breaks in the DNA is compromised.

### *cycG* mutants are hypersensitive to genotoxic stress

**Figure 2:**
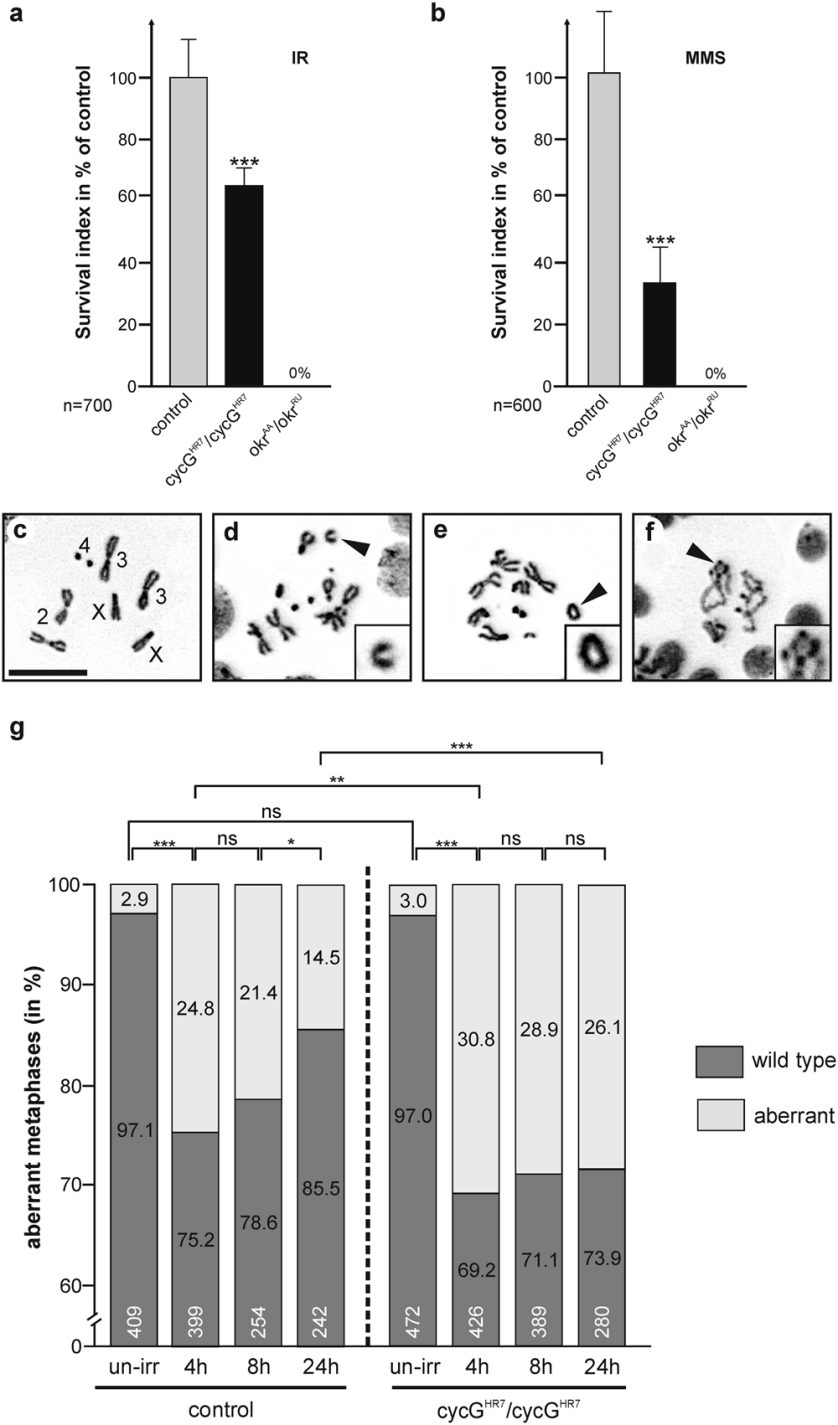
c*ycG* mutants are sensitive to genotoxic stress. **(a,b)** Surviving genotoxic stress. Wild type control (Oregon-R), *cycG* ^*HR7*^ and *okr* ^*AA*^ */okr* ^*RU*^ mutant larvae were exposed to (**a**) IR-stress (40 Gy) or to (**b**) MMS (final ~2 mM). The survival index was determined as fraction of treated vs. untreated flies emerging from larvae and is given as % of the wild type control. The experiments were done in duplicate or triplicate (n = total number of animals for each genotype analysed in the assay); standard deviation is given and significance determined by Student’s T-test (***p < 0.001). **(c–f**) Examples of metaphases in larval neuroblasts un/exposed to IR-stress (12.5 Gy). (**c**) Wild type metaphase. Chromosomes are labelled. Scale bar: 10 μm in all panels. (**d**–**f**) Examples of aberrant metaphases. Arrowheads point to examples (see also insets): chromosome breaks (**d**), single telomere fusion (**e**) or multifusions (**f**). (**g**) Frequency of normal (dark grey bars) and aberrant (light grey bars) metaphases in wild type and *cycG* ^*HR7*^ */cycG* ^*HR7*^ mutant neuroblasts prepared from un-irradiated (un-irr) or irradiated animals. Time point of preparation after irradiation is given underneath the bars in hours, sample size (n) within the bars. Statistical significance was determined with Student’s T-test (ns, not significant; *p < 0.05; **p < 0.01; ***p < 0.001). Compared to control, *cycG* ^*HR7*^ mutant neuroblasts show a higher incidence of chromosomal aberrations in response to irradiation (12.5 Gy), and take considerably longer to recover from IR-stress.

In order to narrow down the role of CycG in sustaining genome stability, we next analysed *cycG* mutants’ sensitivity towards ionizing irradiation (IR) or the DNA damaging agent methyl methanesulfonate (MMS). Both genotoxic stressors directly or indirectly generate DNA single-stranded or double-stranded breaks thereby enforcing a DNA damage response (overview^1,2^). To this end homozygous *cycG* ^*HR7*^ mutant larvae were exposed to 16 Gy IR or to food containing 2 mM MMS, and compared with likewise treated wild type Oregon-R or *okra* (*okra* ^*AA*^/*okra* ^*RU*^) mutant animals. *okra* mutants served as positive control as they are sensitive towards a variety of genotoxic stressors^19,20^. The survival index, i.e. percentage of flies emerging from treated versus untreated larvae was determined for each genotype and related to the control. We found that *cycG* ^*HR7*^ mutants were sensitive to IR with about 60% survival rate of the control, but not as sensitive as the *okra* mutants with no survivors (Fig. 2a). MMS exposure uncovered an even higher sensitivity of the *cycG* ^*HR7*^ mutants with only about 30% survival rate relative to wild type control, but again lesser compared to *okra* mutants (Fig. 2b). These data show that CycG is important for a DNA damage response not only in meiotic but also in somatic tissue. In meiotic tissue, CycG is associated with the 9-1-1 complex^16^, comprising Rad9, Rad1 and Hus1 proteins, which plays a key role in DNA integrity surveillance and damage response^21,22^. We were, however, unable to co-precipitate CycG protein together with Rad9 and Rad1 or BRCA2 in irradiated flies (Supplementary Fig. S2), unlike in female germ cells^16^. This suggests that in somatic cells CycG is not involved in DSB sensing but rather conveys chromosomal stability by protecting chromosomes and/or by assisting in the rapid repair of damaged DNA.

### Increased incidence of mitotic abnormalities after irradiation in *cycG* mutant larval brain cells

To further address the role of the *cycG* gene in chromosome stability, we determined the sensitivity of *cycG* ^*HR7*^ mutants to the induction of chromosomal breaks by irradiation. IR-induced chromosome breaks can be detected in mitotic larval neuroblasts as chromosomal aberrations like fragmentation or telomere fusions^23^. To this end, we examined DAPI-stained preparations of larval brains of either wild type or *cycG* ^*HR7*^ mutants irradiated with 12.5 Gy, and determined the frequency of chromosomal aberrations in a time course of 4, 8 and 24 hours after irradiation. In un-irradiated larvae of either homozygous *cycG* ^*HR7*^ mutants or wild type control, less than 3% of metaphase chromosomes displayed aberrations (Fig. 2c,g). In contrast, 4 hours after IR-treatment, about 30% of *cycG* ^*HR7*^ mutant and 25% of wild type metaphases disclosed damaged chromosomes on average (Fig. 2d–g). This number declined over time, however, to a lesser degree and therefore much more slowly in *cycG* ^*HR7*^ mutant neuroblasts (Fig. 2g). While approximately 14% of the analysed metaphases have defects at 24 hours after irradiation in the wild type, the number is nearly doubled in the *cycG* ^*HR7*^ mutants where about 26% of the analysed metaphases display aberrations, implying a retardation and/or erroneous DNA repair in the absence of CycG.

### CycG is not a transcriptional target of p53 but physically interacts with p53 protein

**Figure 3:**
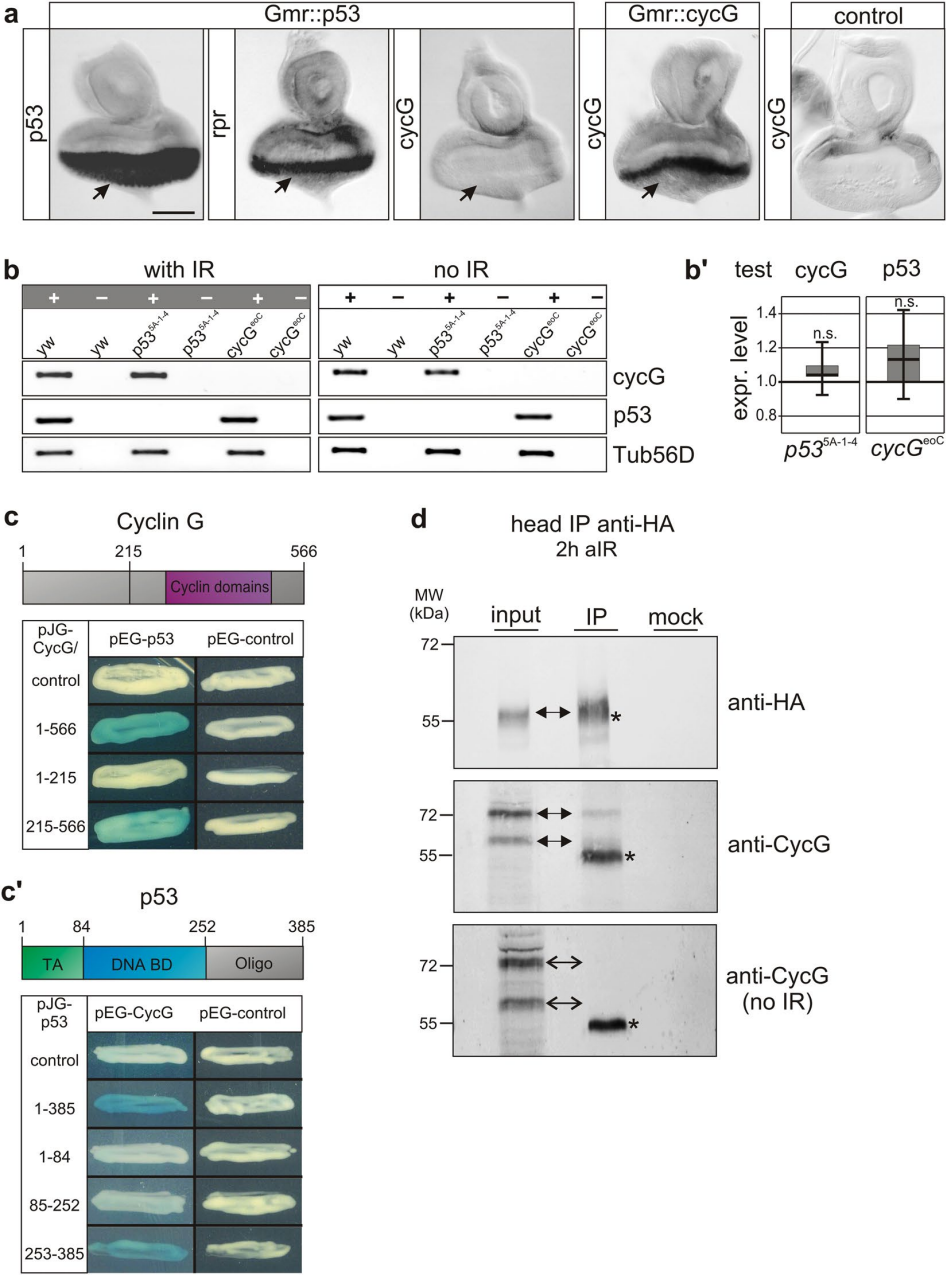
CycG is not a transcriptional target of p53 but associates with p53 protein. **(a**) Eye imaginal discs with UAS-*p53* overexpression driven by *Gmr*-Gal4 were hybridized with the probe indicated, i.e. either *p53* to confirm *p53* expression (arrow), *reaper* (*rpr*) as a known target gene which is indeed turned on (arrow), or *cycG* which does not respond to p53 overexpression (arrow). Quality of the *cycG* probe was monitored on *Gmr*::*cycG* eye imaginal discs and on control discs. Size bar, 50 μm in all panels. (**b**) RT-PCR analyses for *p53*, *cycG* and *beta-tubulin (Tub56D*) transcripts from homozygous adults of the respective genotype with or without irradiation (40 Gy). Reactions were performed with (+) and for control without (−) reverse transcriptase. *cycG* mRNA is present in *p53* mutants and vice versa, independent of irradiation. **(b’)** Expression levels of *cycG* in *p53* ^*5A-1-4*^ homozygotes (left panel) and of *p53* in *cycG* ^*eoC*^ homozygotes (right panel) were quantified by qRT-PCR; no significant differences (n.s.) were detected compared to expression levels of control flies (*y* ^*1*^ *w* ^*67c23*^) (p-value 0.461 and 0.472, respectively). As reference genes to *cycG* (0.98), *βTub56D* (0.98) and *eRF1* (0.98) were used; and *cyp33* (0.95) and *Tbp* (0.93) as reference for *p53 (0.93)*. Target efficiency (given above in parentheses) was taken into account for the relative quantification^51^. **(c,c’)** Yeast two-hybrid interaction assays reveal physical interaction between Cyclin G and p53 protein, as visualized by blue yeast colonies activating the lacZ reporter. Above a scheme of Cyclin G (**c**) and p53 (**c’**) depicts relevant protein domains. Conserved Cyclin domains (AS 287–500, magenta) are relevant for interaction with p53 protein. The p53 protein contains a transactivating domain (TA green, AS 1–84), a DNA-binding domain (DNA BD blue, AS 85–252), and an oligomerization domain (Oligo grey, AS 253–385) which is relevant for CycG binding. Empty vectors served as controls. (**d**) CycG and p53 proteins can be co-precipitated after IR *in vivo*. HA-tagged p53 proteins were immunoprecipitated (IP) from head extracts (*Gmr*-Gal4 UAS-*CycG*::UAS-*p53_3xHA*) 2 hrs after IR using anti-HA antibodies (upper box, arrow). The input lane contained 10% of the protein extract used for the IP. No antiserum was used as mock control. CycG protein can be co-precipitated when flies were irradiated (middle box, arrows), but not without irradiation (lower box). The asterisk labels unspecific IgG signals. Size is given in kDa. Uncropped blots are shown in Supplementary Figure S3.

Presumably, CycG relies on additional partner(s) to pursue its role in safeguarding DNA in somatic tissue. One of the most central players in the control of events following DNA damage is p53. The single *Drosophila p53* gene, for example, was shown to mediate the response to a multitude of stressors by triggering the transcriptional activity of different target genes^7^. So far we know that *Drosophila* CycG is involved in DNA damage recovery after IR-stress, similar to its vertebrate counterpart cyclin G1 (Ccng1)^24^. Interestingly Ccng1, one of the two mammalian cyclin G homologues, is a transcriptional target of p53^25^. Here, Ccng1 together with Mdm2 is involved in p53 negative regulation^10,11^. To elucidate if a comparable scenario also exists in *Drosophila* we addressed the p53-dependence of *cycG* transcription. To this end we overexpressed p53 specifically in larval eye imaginal discs (*Gmr*-Gal4; UAS-*p53*), sufficient to induce expression of the p53 target gene *reaper (rpr)* ^13^ (Fig. 3a). Accumulation of *cycG* transcripts, however, was not observed upon p53 overexpression (Fig. 3a). We wondered whether a response of *cycG* expression to p53 may depend on genotoxic stress, which we analysed by RT-PCR. Expression of *cycG* and *p53* was analysed in wild type and null mutant *p53* ^*5A–*1*–4*^ animals, unchallenged or irradiated with 40 Gy. No apparent difference was observed in *cycG* expression in *p53* ^*5A-*1*-4*^ null mutants versus control, indicating that the transcriptional activation of *cycG* in *Drosophila* is independent of p53 unlike in mammals (Fig. 3b,b’). Moreover, no changes were observed upon IR-stress (Fig. 3b). Also, *p53* transcription was unaffected in *cycG* ^*eoC*^ mutants (Fig. 3b,b’).

Though no transcriptional interdependence exists between *p53* and *cycG* in *Drosophila*, interactions at the protein level remained to be tested. Interaction between *Drosophila* p53 and CycG proteins was assayed in a yeast two-hybrid assay revealing a robust binding between the two proteins (Fig. 3c,c’). We were able to narrow down the p53-interaction domain in CycG to the Cyclin domains, and to the C-terminal part of p53 containing the oligomerization domain (Fig. 3c,c’). To test for *in vivo* interactions, we performed immunoprecipitation with an epitope-tagged version of p53 (UAS-*p53_*3xHA) overexpressed during eye development using *Gmr*-Gal4. Indeed, we were able to co-precipitate p53-HA tagged protein together with concurrently overexpressed CycG (Fig. 3d), however only upon IR-stress and not in unstressed animals. Together, these data clearly reveal that CycG and p53 proteins physically interact, suggesting that CycG may directly modulate the activity of p53 during DSB repair.

### Genetic interactions between *cycG* and *p53*

**Figure 4:**
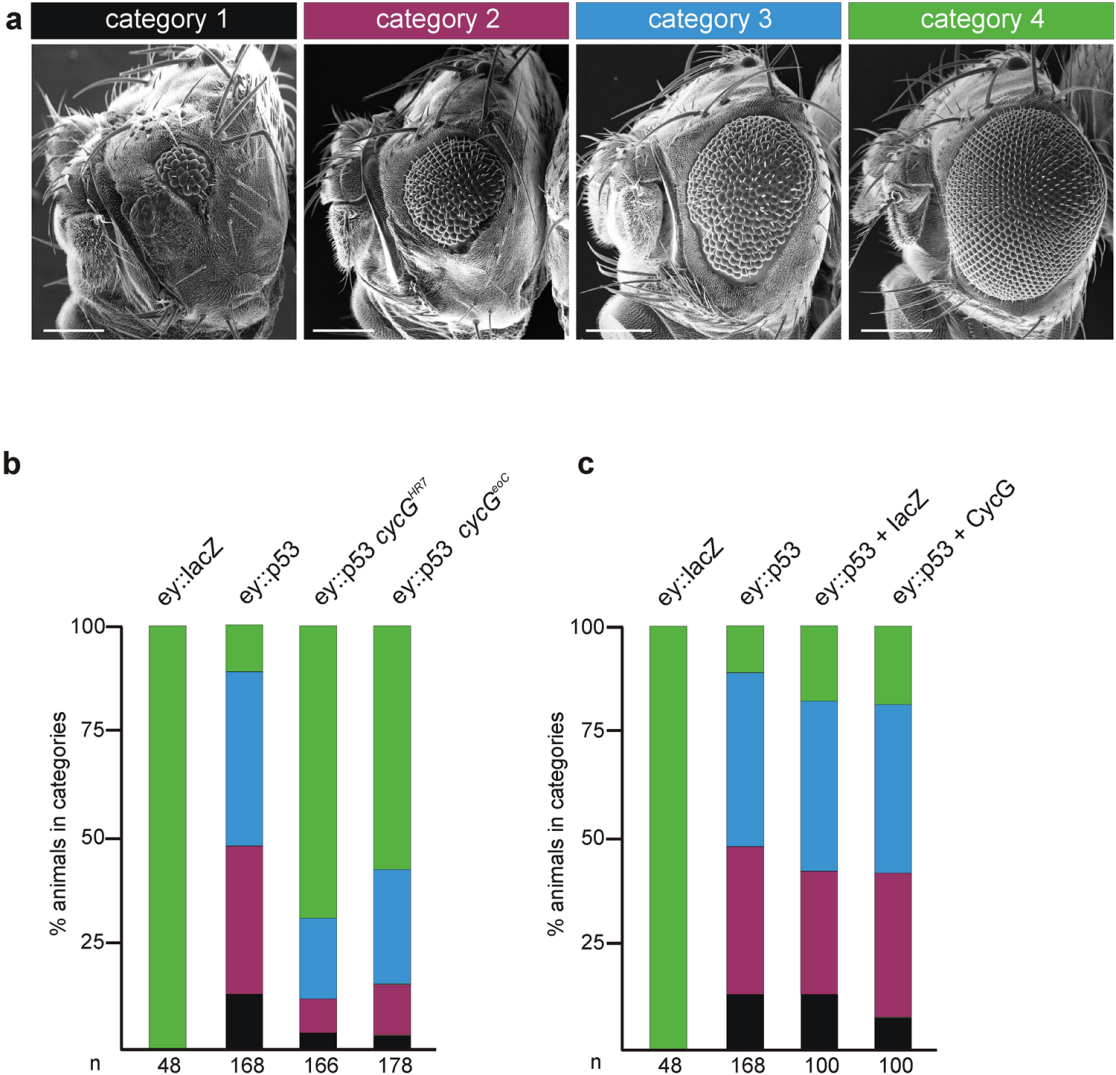
c*ycG* mutants ameliorate the effects of p53 overexpression in the eye. **(a**) Overexpression of UAS-*p53* with *ey*-Gal4 impedes eye development. Effects on eye size were grouped into four categories: 1 (black): no eye/pinhead eye; 2 (magenta): small eye; 3 (blue): smaller than wild type; 4 (green): wild type appearance. Scanning electron micrographs show examples. Size bar, 200 μm. (**b**) *ey::p53* overexpression in the background of homozygous *cycG* mutants, genotypes are indicated. Eye phenotypes of the respective flies were categorized and fractions colour-coded as above. Genotypes are: UAS-lacZ/+; *ey*-Gal4/+control, UAS-*p53*/+; *ey*-Gal4/TM6B, UAS-*p53*/+; *ey*-Gal4 *cycG* ^*HR7*^/*cycG* ^*HR7*^, UAS-*p53*/+; *ey*-Gal4 *cycG* ^*eoC*^/*cycG* ^*eoC*^. (**c**) Combined overexpression of p53 and CycG in the eye anlagen; genotypes are indicated. Phenotypes were categorized as above. Genotypes are: UAS-*lacZ/*+*; ey*-Gal4/+control, UAS-*p53*/+; *ey*-Gal4/TM6B, UAS-*lacZ*/+; UAS-*p53/*+; *ey*-Gal4/TM6B, UAS-*p53*/UAS*-CycG*; *ey*-Gal4/TM6B. n: total number of analysed eyes is given below each column.

According to our hypothesis CycG may be an auxiliary factor of p53 in DNA damage response. In this case we would expect that p53 activity is modulated by *cycG* gene dose which should be uncovered by genetic interactions. Previous studies have shown that ectopic p53 expression affects *Drosophila* eye development: smaller adult eyes with a disturbed ommatidial patterning result from growth and differentiation defects^26^. This phenotype develops when *p53* is constantly induced in cells anterior to the morphogenetic furrow, i.e. within dividing cells. If p53 activity depends on functional CycG in this developmental context, the effects may be modified in a *cycG* mutant background. To test this assumption, the eye phenotype of flies overexpressing p53 (*ey*::*p53*) was investigated in the presence or absence of CycG, using two different *cycG* null alleles *cycG* ^*HR7*^ and *cycG* ^*eoC*^ ^16,27^. The adult progeny was subdivided into four different categories representing the severity of eye size reduction (Fig. 4a). As severity frequently differs between left and right eye, the eyes were scored separately. As a consequence of ectopic p53 expression in the developing eye disc, nearly half of the adult eyes were severely reduced in size (‘pinhead size’) or were considerably smaller, representing categories 1 or 2 (Fig. 4a,b). In total, about 90% of all *ey::p53* eyes were smaller than wild type (Fig. 4b,c). The p53 mediated growth defects were clearly ameliorated in the absence of CycG: in the *cycG* mutant background more than half of the eyes had a wild type size, and the most severe categories were rarely observed, independent of the *cycG* allele used (Fig. 4b). As we have no indication that transcriptional regulation of *ey* is under the control of CycG (Supplementary Fig. S4), we conclude that CycG supports p53 activity at the protein level. We expected that a combined overexpression of p53 with CycG may enhance the p53 eye phenotype, which was however not observed (Fig. 4c), suggesting that CycG requirement for p53 activity is not strictly dose sensitive. Rather CycG appears to be a limiting factor for p53 activity.

**Figure 5:**
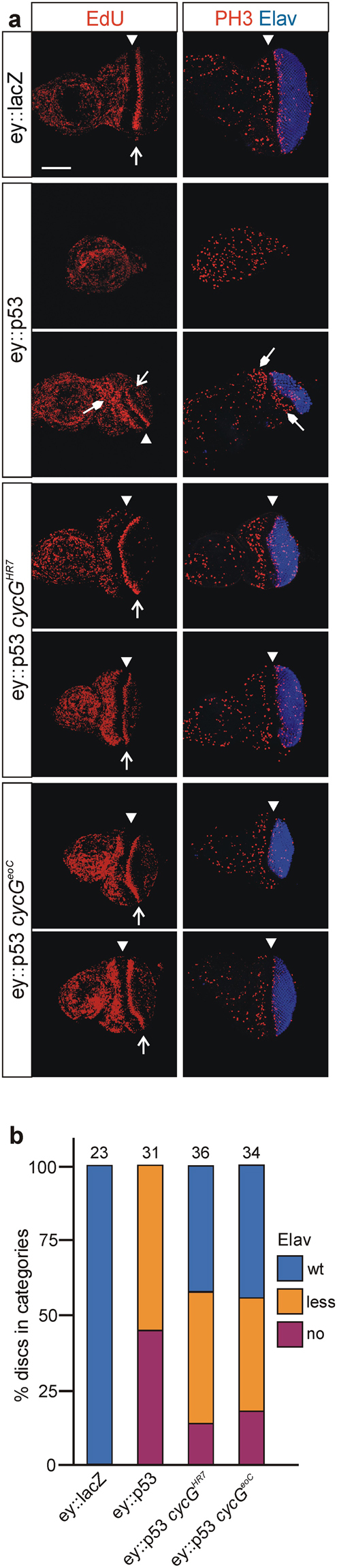
Rescue of p53 overexpression defects occurs already in eye discs. **(a**) Antibody staining of third instar larval eye-antennal discs reveals differences in size of the eye field. DNA-synthesis (EdU-labeling, left column) and cell division (early mitosis marker PH3, right column) is highlighted in red and photoreceptor differentiation (Elav neuronal marker, right column) in blue. The morphogenetic furrow is marked with an arrowhead. Open arrows point to the second mitotic wave. For each mutant genotype, examples for a strong and a weak phenotype are shown. Overexpression of UAS-*p53* with *ey*-Gal4 results in a strongly reduced eye field as visualized by Elav which is decreased or even absent. A weak phenotype is characterized by remains of proliferating eye tissue (closed arrows). Tissue loss is ameliorated in the background of *cycG* mutants, either *cycG* ^*HR7*^ or *cycG* ^*eoC*^, and the discs develop an almost normal eye field. (**b**) The size of the eye field was categorized based on Elav expression as similar to wild type (wt), as clearly smaller than wild type (less) or as absent (no), and discs of the given genotypes were grouped into the respective categories. The number of analysed discs is given above the columns. Eye field size is significantly different between control and all other genotypes, as well as between *ey::p53* and *ey::p53* in any *cycG* mutant background as determined by ANOVA two-tailed Tukey-Kramer approach (***p < 0.001). Blue: Elav staining similar to wild type (wt); orange: reduced Elav field (less); magenta: no Elav staining (no). Genotypes are: UAS-*lacZ*/+; e*y*-Gal4/+ control, UAS-*p53*/+; *ey*-Gal4/+, UAS-*p53*/+; *ey*-Gal4 *cycG* ^*HR7*^/*cycG* ^*HR7*^, UAS-*p53*/+; *ey*-Gal4 *cycG* ^*eoC*^/*cycG* ^*eoC*^.

Adult eye phenotypes reflect the whole developmental process, and a large part of tissue loss may be attributed to increased cell death in pupal stages although overexpression was restricted to the proliferating part of the tissue. To address the specificity of CycG activity regarding p53 mediated growth defects, we analysed the effects at the time of occurrence, i.e. directly in the eye imaginal discs. Moreover, we monitored not only the apparent tissue loss but also influences on cell proliferation. In the developing eye disc, proliferating cells can be visualized by EdU- and Phospho-Histone H3 (PH3) labelling, detecting either cells in the S-phase or mitosis of the cell cycle. Cells anterior to the morphogenetic furrow are vividly proliferating, whereas cells in the posterior part start their differentiation program towards photoreceptor fate and are assembled into ommatidia, visualized by the Elav marker. About 50% of the analysed *ey::p53* eye-antennal discs lacked the posterior part and showed no Elav expression, whereas in the other half the Elav positive part was much smaller (Fig. 5a,b). EdU and PH3 staining, however, appeared similar to wild type in the remaining tissue. In contrast, most of the eye discs from *ey::p53 cycG* mutant animals resembled wild type, using either *cycG* ^*HR7*^ or *cycG* ^*eoC*^ allele. Almost 90% had an Elav positive compartment, i.e. differentiating ommatidia, which was wild type in size in about a third of the discs (Fig. 5a,b). Again, no influence on either EdU or PH3 staining was apparent suggesting that the loss of CycG has no primary effect on cell division. Taken together, our data imply that CycG is a positive mediator of p53 activity also in this developmental context.

### DNA repair processes and initiation of apoptosis are delayed in wing discs of *cycG* mutants similar to *p53* mutants

**Figure 6:**
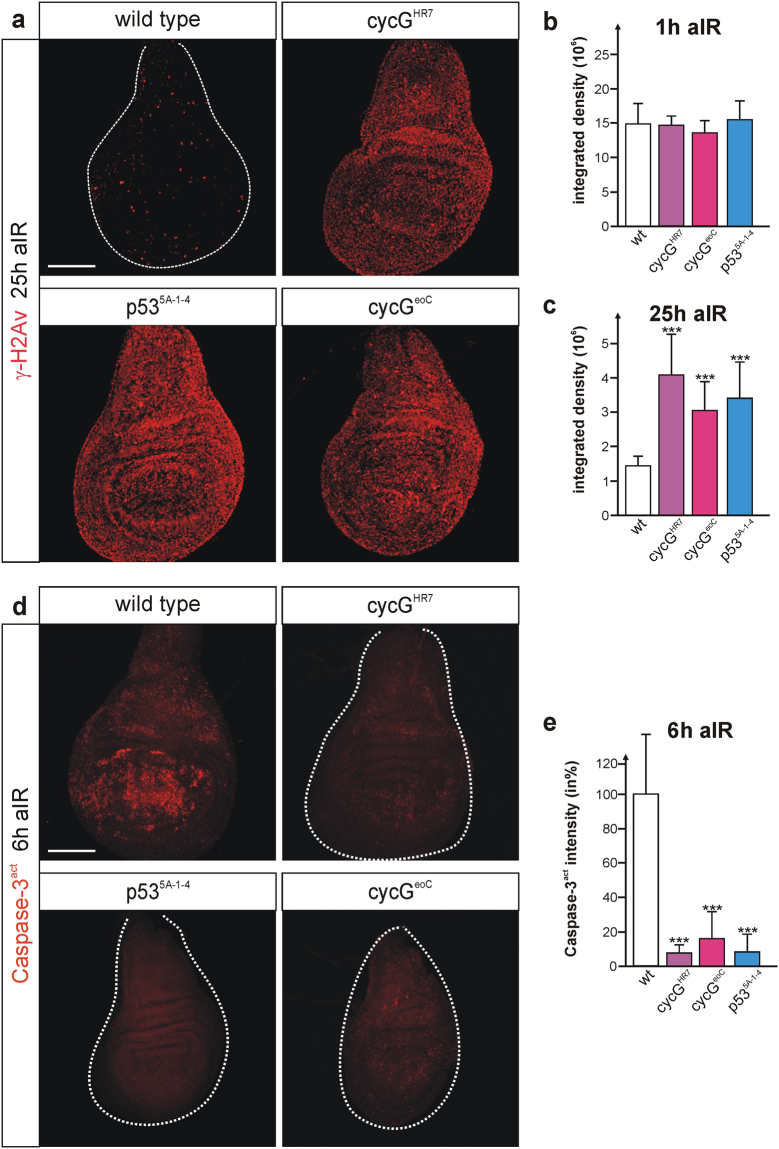
DNA damage response is similarly altered in *p53* and *cycG* mutant discs. Larvae of the given genotype were irradiated (40 Gy) and analysed at the given time (aIR, after irradiation). (**a**) γ-H2Av signals mark foci of damage response; signals are hardly detected in wild type wing discs that have mostly completed repair at 25 hrs after IR. In contrast, γ-H2Av signals are still elevated in *cycG* ^*HR7*^ or *cycG* ^*eoC*^ mutant wing discs. Persistent signals are similarly observed in *p53* mutant wing discs (*p53* ^*5A-1-4*^). (**b,c**) Quantification of γ-H2Av signals was performed by measuring integrated density in 9 to 11 wing discs of each genotype either 1 h (b) or 25 hrs (c) after irradiation using *ImageJ*. Standard deviation is shown; ***p < 0.001 according to Student’s T-test. (**d**) Anti Caspase-3^act^ staining of wing discs 6 hrs after irradiation revealed a robust induction of apoptosis in the wild type, whereas cell death was barely detectable in *cycG* or *p53* mutant discs. (**e**) Quantification of Caspase-3^act^ signals by measuring integrated density in 13–15 wing discs of the respective genotype using *ImageJ*. Standard deviation is shown; ***p < 0.001 according to Student’s T-test. Size bar represents 100 μm in all panels A and D.

DNA damage repair is a highly conserved cellular response involving activation of p53. In *Drosophila*, p53 not only instructs apoptotic elimination of irreversibly damaged cells but is also required for DNA repair and the maintenance of regenerative potential^13,28,29^. One of the first events that allows to visualize DNA damage is phosphorylation of the histone variant H2Av (γ-H2Av) in *Drosophila*, which helps to recruit further DNA repair proteins to the damaged site^3,5^. γ-H2Av can be traced *in vivo* with respective antibodies, and is detected at discrete foci of DSB sites which disappear when DNA repair processes are completed. Third instar larvae mutant for either *cycG* allele *cycG* ^*HR7*^ or *cycG* ^*eoC*^, or mutant for the *p53* null allele *p53* ^*5A–*1*–4*^, were irradiated with 40 Gy, and γ-H2Av accumulation was monitored in wing imaginal discs relative to control (Fig. 6a–c). No differences were seen between mutants and control one hour after irradiation, demonstrating that sensing and initiation of DNA damage repair are unaffected by a loss of CycG (Fig. 6b). In contrast, γ-H2Av foci still persisted 25 hours after IR-stress in the *cycG* mutants, at a time when wild type cells had completed the repair process (Fig. 6a,c). A similar result was seen also for the *p53* mutant, as described earlier^29^ (Fig. 6a–c). Moreover, cell death induction was comparably hampered in *cycG* and *p53* mutant discs: six hours after irradiation a robust induction of apoptosis was observed in the wild type, whereas only rarely detectable in *p53* and *cycG* mutant discs, respectively (Fig. 6d,e). These results provide evidence for an impaired repair process in *cycG* and *p53* mutants alike. We propose that CycG is required to resolve IR-induced DNA damage presumably as co-factor of p53.

DNA damage notably by oxidative stress is a major inducer of aging with p53 being a key coordinator^30^. Accordingly, *p53* mutant flies have a shortened life span compared to the wild type^9^. We compared the life span of the homozygous *p53* ^*5A-*1*-4*^ flies with that of *cycG* ^*HR7*^ and *cycG* ^*eoC*^, wild type Oregon-R and *methuselah mth* ^1^ as example of a long lived stock^31^. As is expected for a common role of p53 and CycG in the context of DNA damage repair, the life span of respective mutants was likewise reduced (Supplementary Fig. S5).

### Activation of the p53 biosensor by IR-stress is reduced in *cycG* mutant germaria

**Figure 7:**
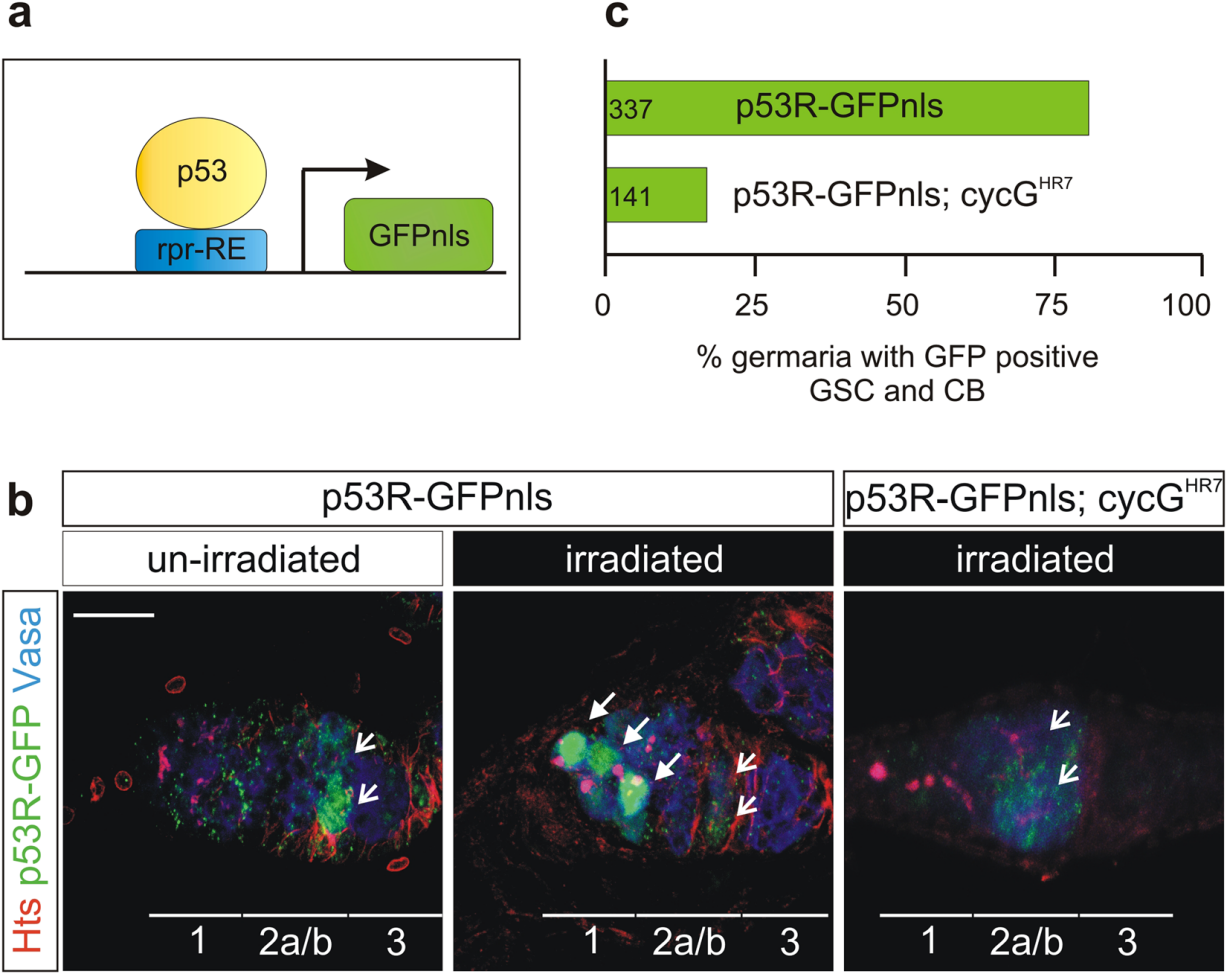
p53 biosensor activity is decreased in *cycG* mutant ovaries. **(a**) The *p53R*-GFPnls p53 biosensor encodes nuclear GFP under the control of a p53-dependent *reaper* response element (*rpr*-RE)^32^. Activation of p53 can be followed *in vivo* by nuclear GFP accumulation as read out. (**b**) Female germaria were stained for Vasa (blue) to detect germ cells, for Hts (red) to detect germline stem cells (GSC) and cystoblasts (CB), and for GFP (green) to visualize p53 activity. In unchallenged ovaria, p53 activity is detected in cysts of region 2a/b undergoing recombination (open arrows). After IR- stress (40 Gy) the p53 biosensor is strongly activated in GSCs and CBs (arrows). In a *cycG* ^*HR7*^ mutant background, p53 activity is barely visible after irradiation. Size bar represents 50 μm. (**c**) Statistical evaluation of *p53R*-GFPnls reporter activity in wild type and *cycG* ^*HR7*^ mutant background after IR challenge. Whereas 80% of the wild type germaria show a strong GFP expression in GSC and CB, only 20% of the *cycG* mutant germaria show this staining. Number of analysed germaria is shown on the left.

Based on our genetic data we favour the idea that in *Drosophila* CycG assists p53 function during DNA damage response. To follow p53 activity directly *in vivo* we made use of a *p53R*-GFPnls biosensor, where p53 activation triggers nuclear GFP expression^32^ (Fig. 7a). This sensor is activated in response to genotoxic stress in the female germline exclusively in germline stem cells (GSC) and their immediate progeny, the cystoblasts (CB)^33^. To determine, whether CycG is required for p53 activity in response to IR-stress, we introduced the *p53R*-GFPnls biosensor into a *cycG* ^*HR7*^ mutant background and measured the GFP reporter activity 24 hours after irradiation (Fig. 7b,c). As described earlier^33^, GFP nuclear localization was detected in region 2a/b of control germaria, where cross-over events are repaired, whereas in response to IR-stress nuclear GFP was detected in gonadal stem cells in more than 80% of germaria (Fig. 7b,c). In the *cycG* ^*HR7*^ mutant background, however, less than 20% of the irradiated germaria showed the expected nuclear GFP accumulation (Fig. 7b,c). These findings strongly corroborate our genetic data and support our hypothesis that CycG is crucial for p53 mediated response to genotoxic stress.

### IR induced p53 biosensor activity is decreased in salivary glands of *cycG* mutants

**Figure 8:**
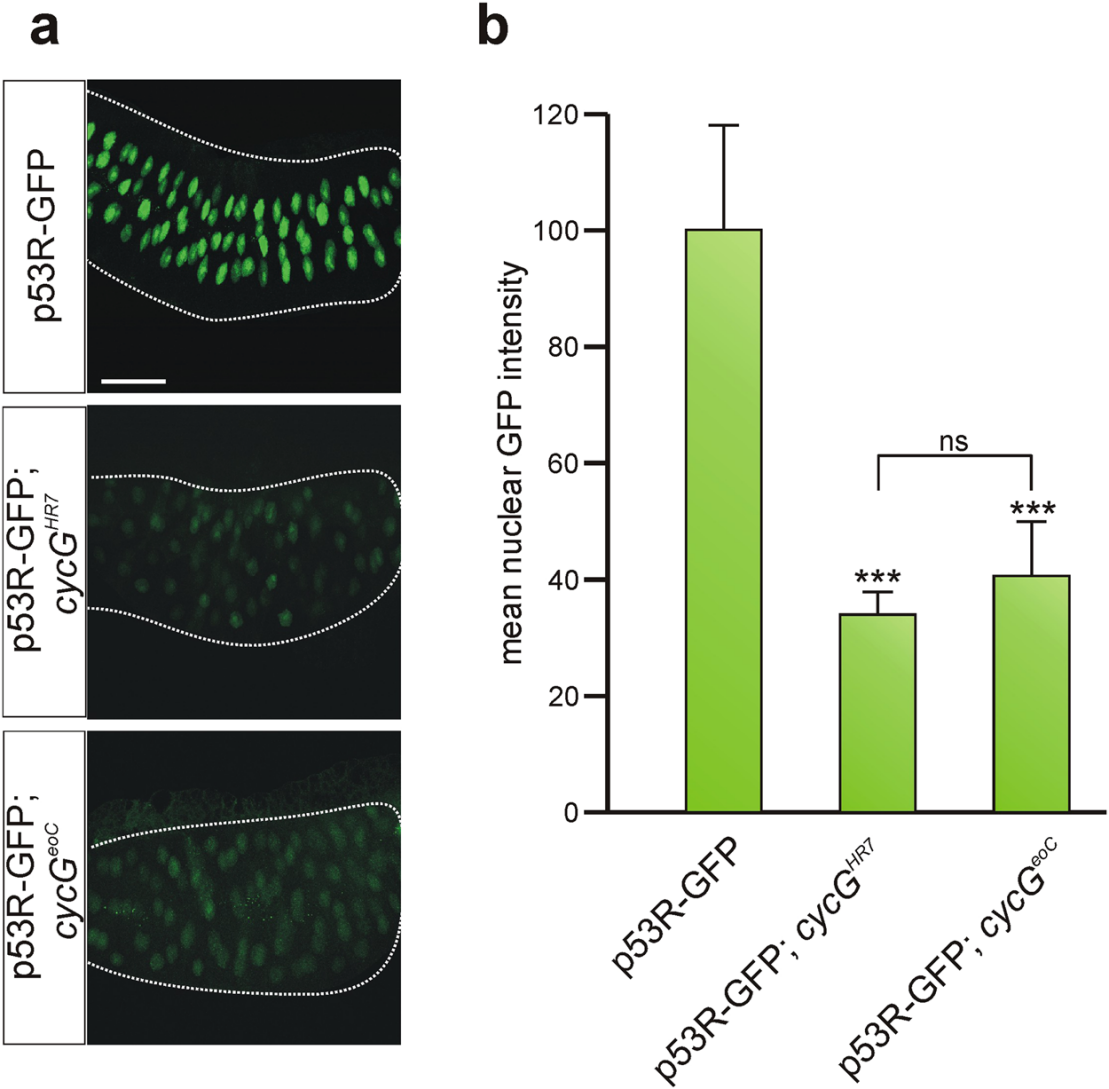
Absence of CycG affects p53 biosensor response to IR-stress in salivary glands. *p53R*-GFPnls reporter activity was analysed in larval salivary glands from either control, *cycG* ^*HR7*^ or *cycG* ^*eoC*^ mutants irradiated with 40 Gy. (**a**) IR-stress results in p53 activation and subsequent expression of nuclear GFP from the p53 biosensor in the control, however to a much lower degree in *cycG* mutant glands. Size bar represents 100 μm in all panels. (**b**) Nuclear intensity of GFP signals was recorded using *ImageJ* by measuring individual nuclei (30–40 per gland; 6–8 glands per genotype). In both *cycG* mutant alleles nuclear GFP staining is reduced to about one-third of the value measured in the wild type. Significance was tested by ANOVA two-tailed Tukey-Kramer approach (***p < 0.001; ns, not significant).

The *p53R*-GFPnls reporter faithfully reports p53 activation after genotoxic stress also in *Drosophila* embryos^32^. To expand our *in vivo* analyses to somatic tissue, we hence exposed *p53R*-GFPnls third instar larvae to irradiation (40 Gy), and monitored GFP expression in polytene nuclei of salivary glands 2 hours after irradiation. IR-stress caused a strong nuclear GFP signal, reflecting IR-induced activation of p53 in our test system (Fig. 8a). In the background of *cycG* mutant alleles *cycG* ^*HR7*^ or *cycG* ^*eoC*^, the intensity of nuclear GFP dropped dramatically to levels of about 30% of the control value (Fig. 8a,b). We conclude that not only in the germline but also in the soma CycG is a pivotal component for either the activation of p53 or for p53 transcriptional activity in response to IR-stress.

## Discussion

Earlier we have shown that *Drosophila* CycG is important for the meiotic recombination checkpoint in the female germline. In *cycG* mutant germaria, DSB repair is delayed, and CycG protein is found in conjunction with the 9-1-1 complex suggesting that it may be involved in DSB sensing^16^. We have now extended our analysis to somatic tissue, where again we note problems in DNA damage repair as detected by persistent γ-H2Av signals in irradiated *cycG* mutants. This indicates that in the absence of CycG, repair of double-strand breaks in the DNA is compromised. Accordingly, *cycG* mutants fail to repair DSBs with the fidelity of wild type, display more chromosomal aberrations upon irradiation, and are hypersensitive to genotoxic stress. We found no evidence for an involvement of CycG in DSB sensing in somatic cells, however. Instead, CycG appears to perform its role through modulating the activity of p53. Since we observe a retardation and/or erroneous DNA repair in the absence of CycG, we propose that CycG is required to resolve IR-induced DNA damage presumably as co-factor of p53. From our genetic data we conclude that CycG is a positive mediator of p53 activity, and indeed mutants in either gene resemble each other not only in life span but also in radiation sensitivity. The physical interaction of CycG and p53, however, strongly suggests that CycG directly promotes p53 activity, regardless of whether it may also regulate downstream or upstream components of the DNA damage repair machinery.

Unlike in vertebrates, *Drosophila cycG* is not under the transcriptional control of p53. Instead we see a robust protein-protein interaction in a yeast two-hybrid assay between p53 and CycG proteins, involving the cyclin repeats of CycG and the tetramerization domain of p53. Direct binding *in vivo*, however, required genotoxic stress. We propose that complex formation, rather than being permanent, occurs only in response to DNA damage and perhaps requires additional factors and/or protein modification/s. With the help of a p53 biosensor we showed that CycG is crucial for p53 mediated transcriptional response to genotoxic stress in the germline as well as in somatic tissue, suggesting that CycG may be involved in the activation or stabilization of p53 itself, or in the assembly of active transcriptional complexes.

The CycG-p53 axis might have been expected given their close interrelationship in the mammalian system. Here, the two cyclin G homologues Ccng1 and Ccng2 have been involved in growth control to genotoxic stress^24,34,35^. Ccng1 but not Ccng2 is a direct transcriptional target of p53^25^. Both are found in complexes with protein phosphatase 2A, and together with Mdm2 Ccng1 is involved in Mdm2 mediated degradation of p53^11,36,37^. As the two mammalian cyclin G proteins appear to act differently on cell proliferation, a lot of work has been invested to understand their respective roles. More recently it was proposed that observed discrepancies may arise from dose dependency of Ccng1^34^. In fact, also *Drosophila* tissues and cells appear to respond differentially to the dose of CycG, as for example overexpression may impact the cell cycle in a dominant negative manner, and RNAi downregulation causes effects different from the gene deletion phenotypes^16,18,27,38,39^. The role of Ccng1 in response to genotoxic stress has been analysed in quite some detail. Here, Ccng1 not only forms a complex with Mdm2, resulting in destabilizing p53. Moreover, it also interacts with ARF, thereby stabilizing and activating p53^40^. It hence has been proposed that Ccng1 is required for a timely and proper response to genotoxic stress, first for the activation of p53 to allow for DNA damage repair, and then for p53 degradation to protect cells from apoptosis that have recovered from the initiating stress^34,40^.

Intensive searches in the *Drosophila* genome failed to uncover Mdm2 or ARF homologues to date. Recently, however, a Mdm2 analogue called Corp has been identified that shares several Mdm2 properties^14^: Corp is a transcriptional target of p53 in response to genotoxic stress, it binds to p53 protein and results in reduced p53 protein levels presumably by proteolytic degradation. Hence, like Mdm2 Corp acts in a negative feed back loop on p53 activity^14^. Whether Corp is likewise inactivated by phosphorylation and/or an ARF-like molecule remains to be shown. Moreover, it will be interesting to see, whether Corp can recruit CycG, and whether PP2A plays any role in its regulation. We know already that *Drosophila* CycG also binds to the PP2A-B’ subunit, similar to the two mammalian CycG proteins^27,36,37,41^. Unlike in vertebrates, however, it acts negatively on PP2A activity by genetic means^27,41^. Despite the similarity of the respective components and the processes they are involved in, there is not a 1:1 conformity when comparing flies and mammals. Perhaps, the manifold feed back loops weaved into the system, obstruct our view and elude genetic analyses. Perhaps, like in mammals^34,40^, *Drosophila* CycG forms protein complexes with disparate activities depending on tissue, cell cycle phase, or phase of response to DNA damage - repair or apoptosis.

## Materials and Methods

### Fly stocks and genetic work

Three null alleles of *cycG* were used, *cycG* ^*HR7*^ ^16^, *cycG* ^*CreD*^ ^18^ and *cycG* ^*eoC*^ ^27^. *cycG* ^*HR7*^ was generated by ends in homologous recombination and carries the *white* ^+^ marker in between two defective *cycG* copies. From this allele, *cycG* ^*CreD*^ was derived lacking the *white* ^+^ marker. *cycG* ^*eoC*^ was generated by ends out homologous recombination. All three alleles are null mutants as judged by molecular analyses and lack of protein expression. They all produce homozygous, sterile offspring at variably low frequency, with *cycG* ^*eoC*^ producing the lowest numbers^16,18,27^. In this work most analyses were performed with the allele *cycG* ^*HR7*^. Flies were reared on standard fly food at 18 °C. Crosses were conducted at 25 °C. The following stocks were used: *mth* ^1^ (BL27896)*, p*53^*5A-*1*-4*^ (BL6815), *okr* ^*RU*^/*CyO* (BL5098)^19^ and UAS-*lacZ* ^42^. They were obtained from the *Drosophila* stock center Bloomington. UAS-*CycG* is described^16^. UAS-Flag-*Rad9* ^20^ and UAS-GFP-*Rad1* ^43^ were obtained from U. Abdu, and *okr* ^*AA*^/*CyO* ^19^ was a gift of T. Schüpbach. *Ey*-Gal4^44^, *Gmr*-Gal4^45^, UASp-*p53* ^46^, and *p53R*-GFPnls^32^, were gifts from U. Walldorf, U. Abdu and J. Abrams. UAS-*p53_3xHA* (#F000091 FlyORF, Zürich, Switzerland) was obtained from the Zurich ORFeome Project. Oregon-R and *y* ^*1*^ *w* ^*67c23*^, respectively served as wild type control. Flies were combined and recombined according to standard genetics and genotypes were verified by PCR. For fly selection, GFP or *Tb* ^*1*^ markers on balancer chromosomes were used. Adult eyes were documented as described earlier^27,47^.

### Lifespan analysis

Freshly hatched flies were collected under mild anaesthesia with CO_2_, and groups of 10 females and 10 males each per vial were collected and reared on standard fly food at 25 °C. Flies were transferred onto fresh food every other day. The number of dead flies was recorded at least every other day. A minimum of 200 flies were analys ed per genotype.

### Genetic reporter assay for monitoring DNA repair efficiency

Crosses were done according to^17^, and flies were kept at 25 °C. Genotypes were: *CyO* Δ2-3/*amos* ^*Tft*^; *cycG* ^*CreD*^/TM3 *Sb* ^*1*^ (derived from BL8201), P{*w* ^*a*^} (gift from J. Sekelsky)^17^ and P{*w* ^*a*^}; *cycG* ^*CreD*^/TM3 *Sb* ^*1*^. The parental crosses were set up in batches of 30, each with 20 virgins and 10 males. In the end approximately 6000 females of the second generation were analysed for each genotype.

### MMS and Ionizing Radiation (IR) treatment

For methyl methanesulfonate (MMS) treatment, parental flies were allowed to lay eggs on apple juice plates with yeast paste over night. At least 100 larvae at early second larval instar were collected for each genotype and transferred into vials with freshly made food containing MMS in a final concentration of about 2 mM (Sigma-Aldrich; St. Louis MO, USA) or without MMS as control. For treatment with IR, second to third instar larvae were collected and irradiated in batches of 100 animals at doses varying from 12.5 Gy (for chromosomal squashes of larval brains), 16 Gy (for determination of adult survival rate) to 40 Gy (for immunohistochemical analyses of larval discs or adult ovaries) using either an Isovolt 255 HS (Richard Seifert & Co. GmbH; Ahrensburg, Germany) at the Max Planck Institute of Intelligent Systems Stuttgart, or an Elektra Versa HD linear accelerator (Elektra Instrument AB; Stockholm, Sweden) at the Marienhospital Stuttgart. Flies were allowed to develop at 25 °C; number of emerging flies was recorded. The survival index of a given genotype was calculated as fraction of treated vs. untreated flies emerging from larvae (given as % of the wild type control). The experiments were done in duplicate or triplicate and a total of minimal 600 animals.

### Neuroblast chromosome squashes

Whole brains of late 3^rd^ instar larvae were dissected in 3 ml 0.7% sodium chloride and, after addition of 6 µl 10 µM colchicine (Serva; Heidelberg, Germany), incubated for 40 min at room temperature. They were next transferred for 9 min into a hypotonic solution of 0.5% sodium citrate and fixed for 5 min in 3% paraformaldehyde dissolved in 50% acetic acid. Brains were squashed on Polysine^TM^ coated slides (Gerhard Menzel GmbH; Braunschweig, Germany) under cover slips coated with Gel Slick^TM^ (AT Biochem; Malvern, Pennsylvania, USA). The slides were frozen in liquid nitrogen, and the cover slips were removed. After several washing steps in TBS (20 mM Tris-HCl pH 7.4, 150 mM NaCl) and TBST (TBS plus 0.5% Triton X-100) the preparations were mounted in Roti^®^-Mount FluorCare with DAPI (Roth; Karlsruhe, Germany). Metaphases were analysed with an Axiophot 2 plus (Zeiss; Göttingen, Germany) coupled to a Power Shot A95 camera (Canon; Krefeld, Germany).

### Protein expression analysis in tissue

Immunostaining of imaginal discs, germaria and salivary glands was done according to standard protocols using the following antibodies: mouse anti-γ-H2Av Unc93-5.2.1 (1:500), mouse anti-Hts 1B1 (1:20), rat anti-Elav 7E8A10 (1:50) and rat anti-Vasa (1:50) (developed by R.S. Hawley, H.D. Lipshitz, G. Rubin and A.C. Spradling & D. Williams, respectively; obtained from the Developmental Studies Hybridoma Bank developed under the auspices of the NICHD and maintained by the University of Iowa, Dept. of Biology, Iowa City, IA 52242); rabbit anti-cleaved Caspase 3 (1:250; Cell Signaling; Germany; called Caspase-3^act^), rabbit anti-GFP (1:100; Santa Cruz Biotech; Dallas, USA) and rabbit anti-Phospho-Histone H3 (PH3) (1:50; Cell Signaling; Germany). For labelling cells undergoing DNA synthesis, the Click-iT EdU Alexa Fluor 488 Imaging Kit (Invitrogen; Eugene, Oregon, USA) was used, and the tissue was treated as described^48^. Anti γ-H2Av staining on larval wing discs was performed 1 h and 25 hrs after irradiation of wandering third instar larvae at 40 Gy. Frequency of remaining DSBs was determined by measuring the integrated density of signals in the wing discs with *ImageJ*. Secondary antibodies with minimal cross-reactivity coupled to DTAF, Cy3 or Cy5 generated in goat were purchased from Jackson Immuno-Research Laboratories (Dianova; Hamburg, Germany). Dissected tissue was embedded in Vectashield (Vector Laboratories; Burlingham, CA, USA) and documented with a Zeiss Axiophot microscope (Carl Zeiss AG; Oberkochen, Germany) coupled to a Bio-Rad MRC1024 scan head using LaserSharp 2000 imaging software. Pictures were assembled with Corel Photo Paint and Corel Draw software.

### Statistical evaluation of the data

Statistical analysis of probes was performed by Student’s T-test or by ANOVA using a two-tailed Tukey Kramer test for multiple comparisons with p-values (p > 0.05 not significant, n.s.), (p < 0.05 significant, *), (p < 0.01 very significant, **), (p < 0.001 highly significant, ***).

### RNA expression analysis

*In situ* hybridization on larval eye discs was performed with digoxygenin labelled DNA probes of *p53*, *rpr* and *cycG* according to standard protocols^49^. To isolate mRNA for RT-PCR, the PolyATract^®^ System 1000 (Promega; Mannheim, Germany) was used. For each sample, 100 mg of adult flies of the respective genotypes was used. Reverse transcription was performed with oligo-dT primers using the ProtoScript^®^II First Strand cDNA Synthesis Kit from New England Biolabs; Frankfurt, Germany. Following primer pairs were used for the gene specific amplification: *cycG* Exon 5/6, upper primer 5′ CTT CAG CAT CGT TTC GGG TAT TCT 3′; lower primer 5′ ATG GGA TAT GCC TAG TGC CAG GAA A 3′; *p53*, upper primer 5′CAA ATA GTC GGT GGC CAC TAC G 3′; lower primer 5′ TGC AAG AAG GCC ATG GGT T 3′; *βTub56D*, upper primer 5′ GAA CCT ACC ACG GTG ACA GCG A 3′; lower primer 5′ GAA GCC AAG CAG GCA GTC GCA 3′. Quantative RT-PCR was performed on three biological replicates of cDNA obtained as above and outlined before^47^ from 20 adult flies of the given genotype. Real time qPCR was conducted with Blue S’Green qPCR Kit (Biozym; Hessisch-Oldendorf, Germany) on 2 µl of cDNA (0.012 µg) using MIC magnetic induction cycler (bms; Pots Point Australia) as described earlier^47^. As internal reference genes, *βTub56D* (PP17563) and *eRF1* (PP11596) were selected for *cycG* expression, and *cyp33* (PP14577) and *Tbp* (PP1556) for *p53* expression, based on variance, Cq values and matching expression profiles; primer pair sequences (in parentheses) are listed at DRSC FlyPrimer bank^50^. Relative quantification of the data was performed with *micPCR* ^©^ software Version 2.6 based on *REST* ^©^^51^, taking target efficiency into account.

### Protein-protein interaction studies

Pair-wise protein interaction studies were performed using yeast two-hybrid assays. CycG constructs have been described^27^. dmp53cDNA^13^ served as template for PCR amplification of p53 DNA segments. Thereby, we introduced *Eco*RI (5′) and *Xho*I restriction sites (3′) for cloning into the pEG202 and pJG4-5 vectors^52^. Yeast two-hybrid studies were done according to standard protocols with EGY40 cells (*Mata, ura3, his3, trp1, leu2*, GAL)^52^.

For co-immunoprecipitations, protein extracts were derived from un/irradiated (40 Gy) flies overexpressing the respective tagged proteins. To this end, 200 adult heads were homogenised 2 hrs after treatment in binding buffer described in^53^. The protein extract was incubated with 40 µl Protein A Sepharose beads (Roche Diagnostic; Basel, Switzerland) for 2 hrs at 4 °C in the presence of either 18 μl rat anti-HA or rabbit anti-Flag (Roche Diagnostic; Basel, Switzerland resp. Cell Signaling; Germany) antibodies. Subsequently, protein A beads were washed several times with binding buffer at 4 °C and loaded on a SDS-PAGE followed by Western blotting. The blots were probed with rat anti-CycG (1:500)^38^, rat anti-HA (1:500; Roche Diagnostic; Basel, Switzerland), mouse anti-Flag (1:100; Cell Signaling; Germany) or mouse anti-GFP (1:500; Santa Cruz Biotechnology; Dallas, USA). Secondary antibodies coupled to alkaline phosphatase were used (1:1000) (Jackson Immuno-Research Laboratories; Dianova; Hamburg, Germany). Two different genotypes were analysed: *Gmr*-Gal4 UAS-*CycG*::UAS-GFP-*Rad1* UAS-Flag-*Rad9* for precipitation with anti-Flag and *Gmr*-Gal4 UAS- *CycG*::UAS-*p53_3xHA* for precipitation with anti-HA.

### Data availability statement

All relevant data are within the manuscript and its supplementary information files.

## Electronic supplementary material

Supplementary Information
