# Supplementary material for: p53 and cyclin G cooperate in mediating genome stability in somatic cells of Drosophila
Source: Sci Rep. 2017 Dec 20;7:17890. doi: 10.1038/s41598-017-17973-z (PMC5738409; doi:10.1038/s41598-017-17973-z)
Supplement: Supplementary file 1 — Supplementary Information [file 41598_2017_17973_MOESM1_ESM.pdf]

Supplementary Information to

p53 and *cyclin G* cooperate in mediating genome stability in somatic cells of *Drosophila*

Fabienne E. Bayer[1,¶], Mirjam Zimmermann[1,¶], Patrick Fischer[1,2], Christian Gromoll[3], Anette Preiss[1]* and Anja C. Nagel[1]*

[1]Institut für Genetik, Universität Hohenheim, Stuttgart, Germany

[2]current address: Institute of Molecular Biology/CMBI, University of Innsbruck, Innsbruck, Austria

[3]Department of Medical Physics, Marienhospital Stuttgart, Stuttgart, Germany

[¶:] These authors contributed equally to this work.

*: corresponding authors

e-mails: anja.nagel@uni-hohenheim.de / a.preiss@uni-hohenheim.de

Supplementary Figure S1: Crossing scheme for P $\{w^a\}$ based assay

Supplementary Figure S2: CycG does not co-precipitate with components of 9-1-1 complex in somatic tissues

Supplementary Figure S3: Uncropped blots shown in Figure 3d

Supplementary Figure S4: *eyeless* expression is not under the control of CycG

Supplementary Figure S5: *cycG* and *p53* mutant flies have a decreased life span

Supplementary Figure S1: Crossing scheme for P {w^a} based assay

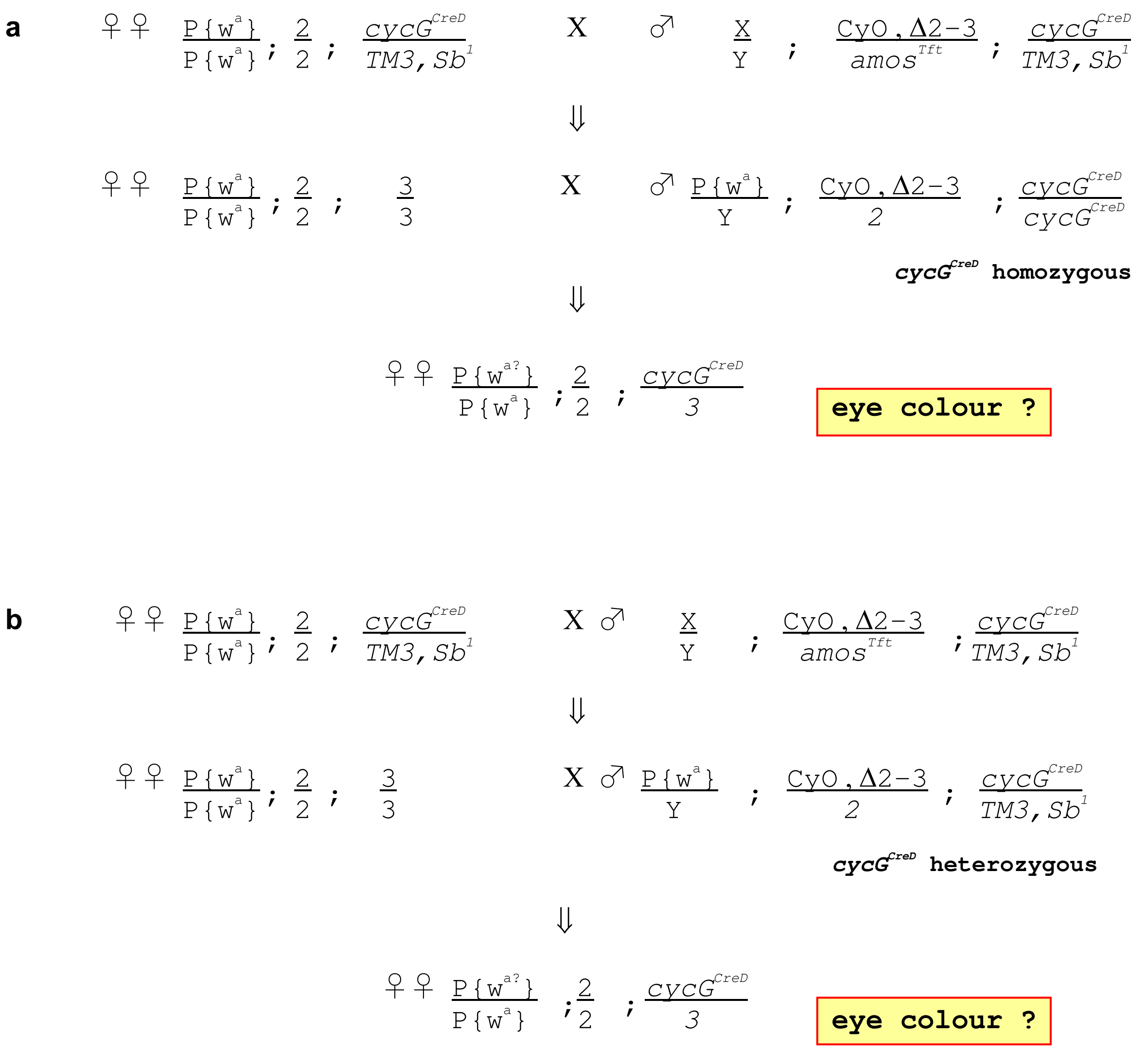

Crossing scheme for recovering DSB repair events following excision of P{w^a} is shown [according to Adams *et al.*, *Science* **299**, 265-267 (2003)]. Mitotic germline repair events were recovered by crossing males harbouring a X-linked copy of P{w^a}, a transposase source {CyO, *Δ2-3/amos*^{Tft}} and either homozygous **(a)** or heterozygous **(b)** *cycG*^{CreD} mutant chromosomes to homozygous *w* P{w^a} virgin females. Female progeny was scored for eye colour, which is indicative for precise (apricot-coloured eyes) or aberrant (yellow or red coloured eyes) DSB repair events.

Supplementary Figure S2: CycG does not co-precipitate with components of 9-1-1 complex in somatic tissues.

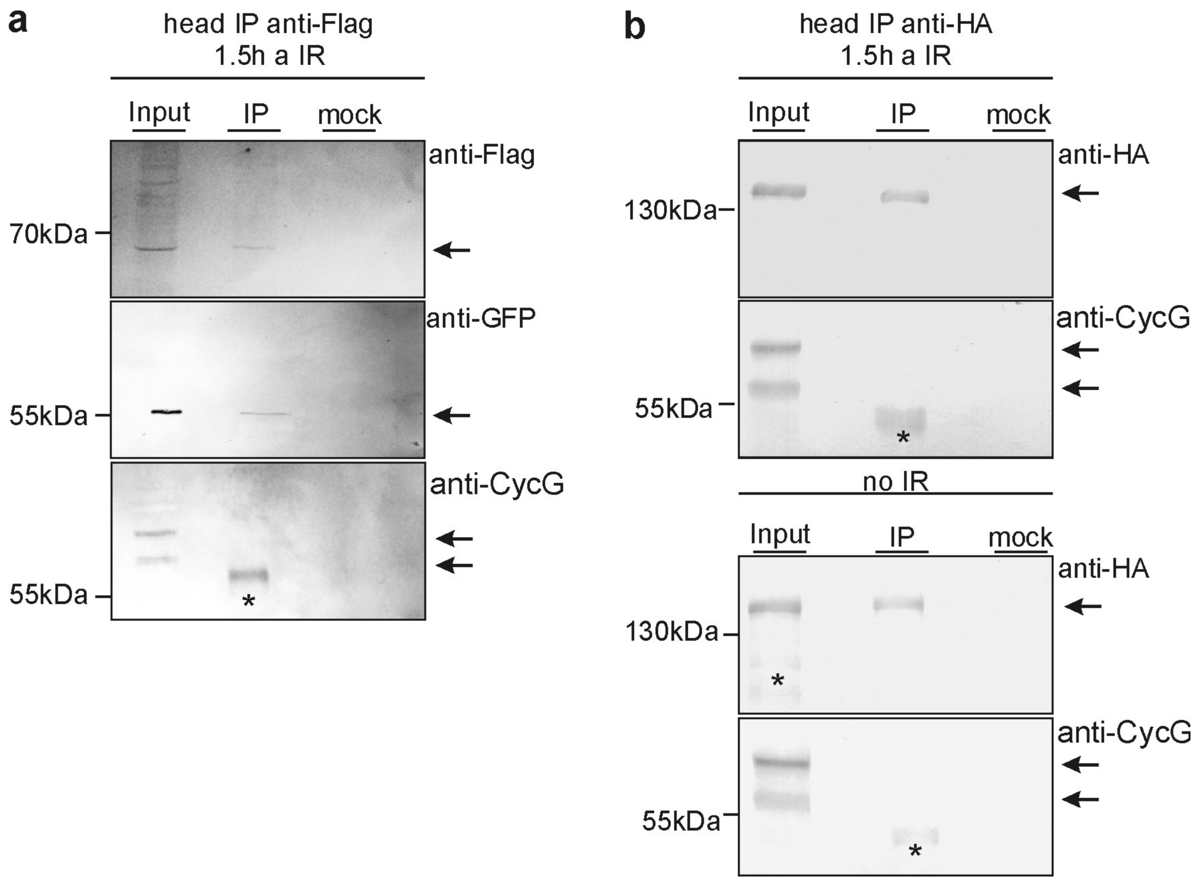

The 9-1-1 complex, comprising Rad9, Rad1 and Hus1, is pivotal to DNA integrity surveillance [Kemp & Sazar, *Curr Biol.* **19**, R733-734 (2009)]. During meiotic DSB response, CycG was found in a complex together with Rad9 and BRCA2 in the female germ line, [Nagel *et al. J Cell Sci.* **125**, 5555-5563 (2012)]. To address the question, whether CycG likewise associates with the 9-1-1 complex or with BRCA2 in somatic tissues upon DNA damage, UAS-CycG was overexpressed concomitantly with UAS-Rad9-Flag and UAS-Rad1-GFP **(a)** or with UAS-BRCA2-HA **(b)** in eye imaginal discs using *Gmr*-Gal4. Emerging flies were irradiated with 40 Gy, and protein extracts derived 1.5 hours later from adult heads. Immunoprecipitation was performed with anti-Flag antibodies to pull down Rad9 **(a)** or with anti-HA antibodies to pull down BRCA2 **(b)**; the input contained 10% of the IP; mock contained no antibody. Complex formation of Rad1 and Rad9 was confirmed, as Rad1-GFP was detected in the Rad9-Flag precipitate, whereas no CycG protein was detected in the complex **(a)**. Likewise no interaction of CycG with BRCA2-HA protein was observed independent of irradiation **(b).** Asterisks indicate unspecific signals.

Supplementary Figure S3: Uncropped blots shown in Figure 3d

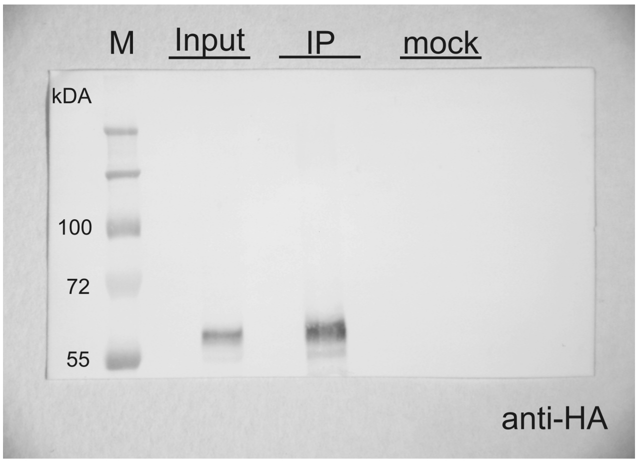

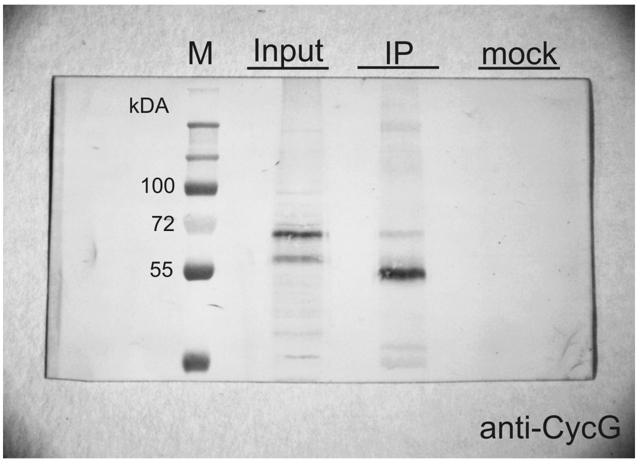

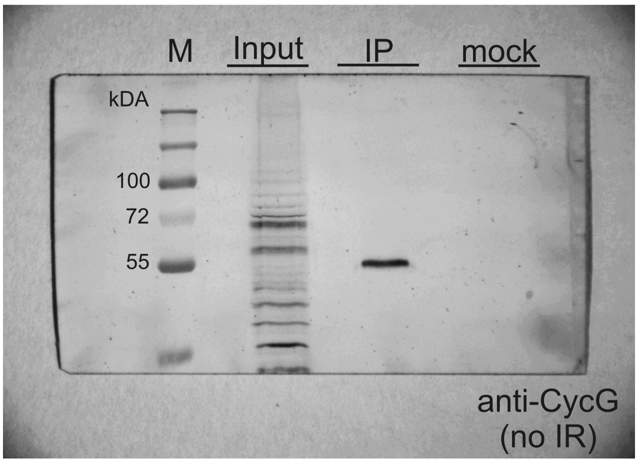

Uncropped blots as shown in Figure 3d.

Upper blot: HA antibodies were used to precipitate p53-HA tagged protein (IP) from head extracts (*Gmr*-Gal4 UAS-*CycG*::UAS-*p53_3xHA*) 2 hrs after IR. Input contained 10% of protein extract; no antiserum was used as mock control; M, prestained protein marker for size control (two prominent bands labelled in kDa).

Middle blot: anti-HA precipitate from irradiated animals as above was probed with anti-CycG antiserum for the presence of CycG.

Lower blot: co-IP as above using unirradiated animals.

Supplementary Figure S4: *eyeless* expression is not under the control of CycG

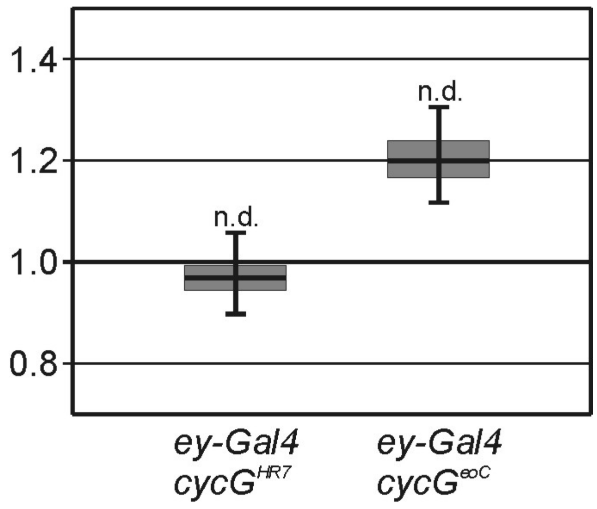

In order to address, whether *eyeless* expression itself is under the control of CycG, expression levels were quantified by qRT-PCR. To this end, Gal4-levels from homozygous *ey*-Gal4 $cycG^{HR7}$ or *ey*-Gal4 $cycG^{eoC}$ flies were compared with those from the control *ey*-Gal4. As reference genes *cyp33* and *Tbp* were used. No significant differences to the *ey-Gal4* control were detected (n.d.; p-value 0.353 and 0.0585, respectively). Efficiencies for *Gal4* (0.90), for *cyp33* (0.94) and for *Tbp* (0.91) were taken into account for the relative quantification according to Pfaffl et al. [*Nucl Acids Res* **30**(9):e36 (2002)]. Data assembly was from three biological replicates; mini-max depicts 95% confidence, median corresponds to expression ratio. Primer pairs for *cyp33* PP14577 and for *TbP* PP1556 were according to DRSC FlyPrimerBank; Gal4 forward AGAGGTATGTGCGCCGTTTCTGT and Gal4 reverse GGGTATTGGGCGATAGTTGCAGA. RNA was derived from 20 adult flies each; for further details, see Materials and Methods.

Supplementary Figure S5: *cycG* and *p53* mutant flies have a decreased life span

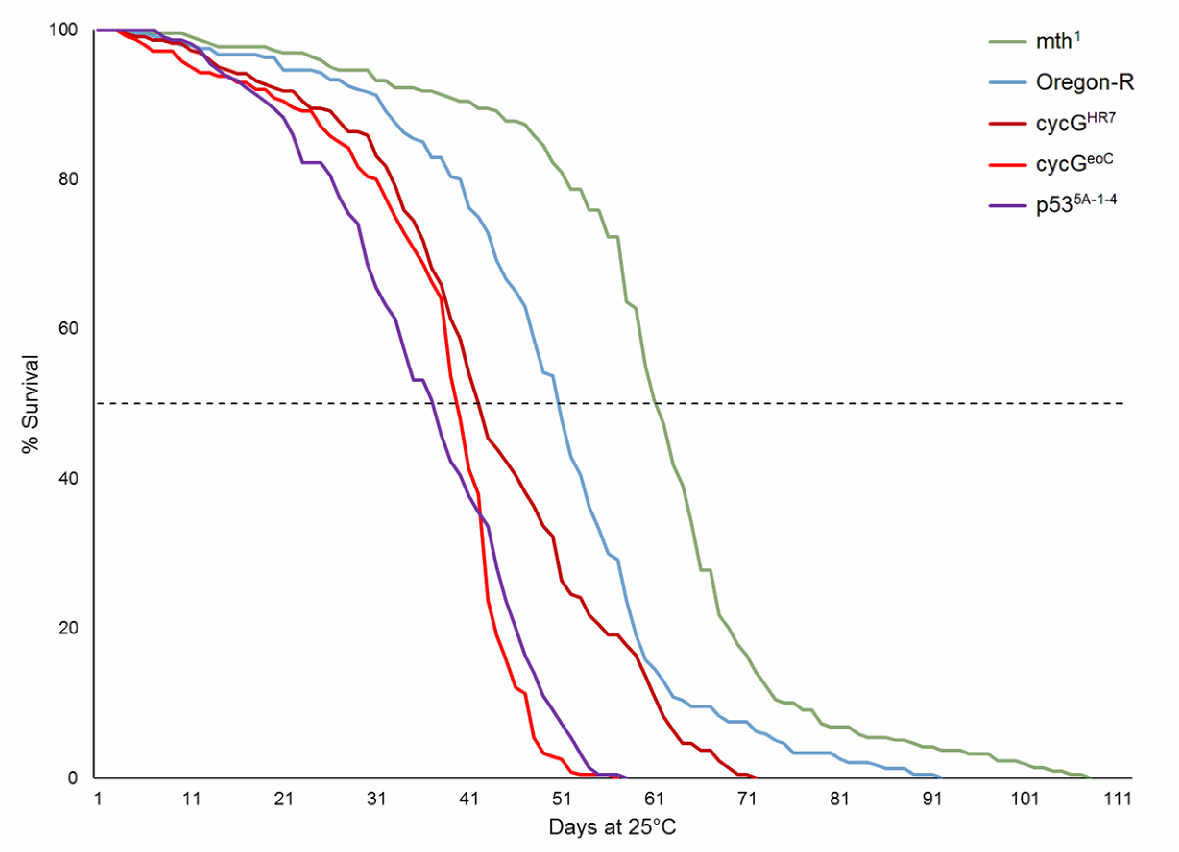

Life span of *cycG* (*cycG^{HR7}*, *cycG^{eoC}*) or *p53* (*p53$^{5A\text{-}1\text{-}4}$*) mutant flies is similarly reduced in comparison to a wild type strain (Oregon-R), whereas *methuselah* (*mth^{1}*) mutants are characterised by a prolonged life span. At least 200 flies for each genotype were analysed in parallel under the same conditions. Dashed line indicates half life time.
